# CT-based evaluation of patient-prosthesis mismatch after transcatheter aortic valve implantation and its influence on outcome

**DOI:** 10.1007/s00392-025-02701-9

**Published:** 2025-06-23

**Authors:** Ramona Schmitt, Nils Ressler, Klaus Kaier, Johannes Brado, Manuel Hein, Martin Soschynski, Dirk Westermann, Franz-Josef Neumann, Philipp Breitbart, Philipp Ruile

**Affiliations:** 1 Department of Cardiology and Angiology, Faculty of Medicine, Medical Center, University of Freiburg, University of Freiburg, Südring 15, 79189 Bad Krozingen, Germany; 2 Institute of Medical Biometry and Statistics, Faculty of Medicine and Medical Center, University of Freiburg, Freiburg, Germany; 3 Department of Diagnostic and Interventional Radiology, Faculty of Medicine, Medical Center-University of Freiburg, University of Freiburg, 79106 Freiburg, Germany

**Keywords:** Computed tomography angiography, Patient-prosthesis mismatch, TAVI, Bioprosthetic heart valves

## Abstract

**Background:**

Patient-prosthesis mismatch (PPM) after trans-catheter aortic valve implantation (TAVI) is a risk factor for heart failure and mortality. Assessment of PPM using transthoracic echocardiography (TTE) and presence of hypo-attenuated leaflet thickening (HALT) may lead to overestimation. Our study aimed to assess the incidence of PPM using TTE and CTA after exclusion of patients with HALT and to evaluate predictors analyzing stent geometry.

**Methods:**

444 patients were analyzed. PPM was calculated using the continuity equation from TTE (TTE-PPM) and CTA (CT-PPM). Regression analyses were conducted for the endpoint effective orifice area (EOA) as surrogate for PPM.

**Results:**

Severe PPM was detected in 4.5% using TTE-PPM and in 0.5% using CT-PPM. Body mass index (BMI) was identified as a predictor for a smaller EOA in each model (*p* < 0.001). Using TTE-PPM and CT-PPM, a smaller valve diameter was associated with a smaller effective orifice area (EOA) (*p* < 0.001). Presence of a balloon-expanding valve was associated with a smaller EOA using CT-PPM (*p* = 0.033). Stent geometry did not influence the EOA (*p* > 0.05 each model). EOA did not influence overall survival (*p* > 0.05 each model).

**Conclusions:**

The incidence of severe PPM was very low and only predicted by BMI in each assessment model. Stent geometry did not influence the incidence of PPM. Overall survival was not influenced by a smaller EOA.

**Graphical abstract:**

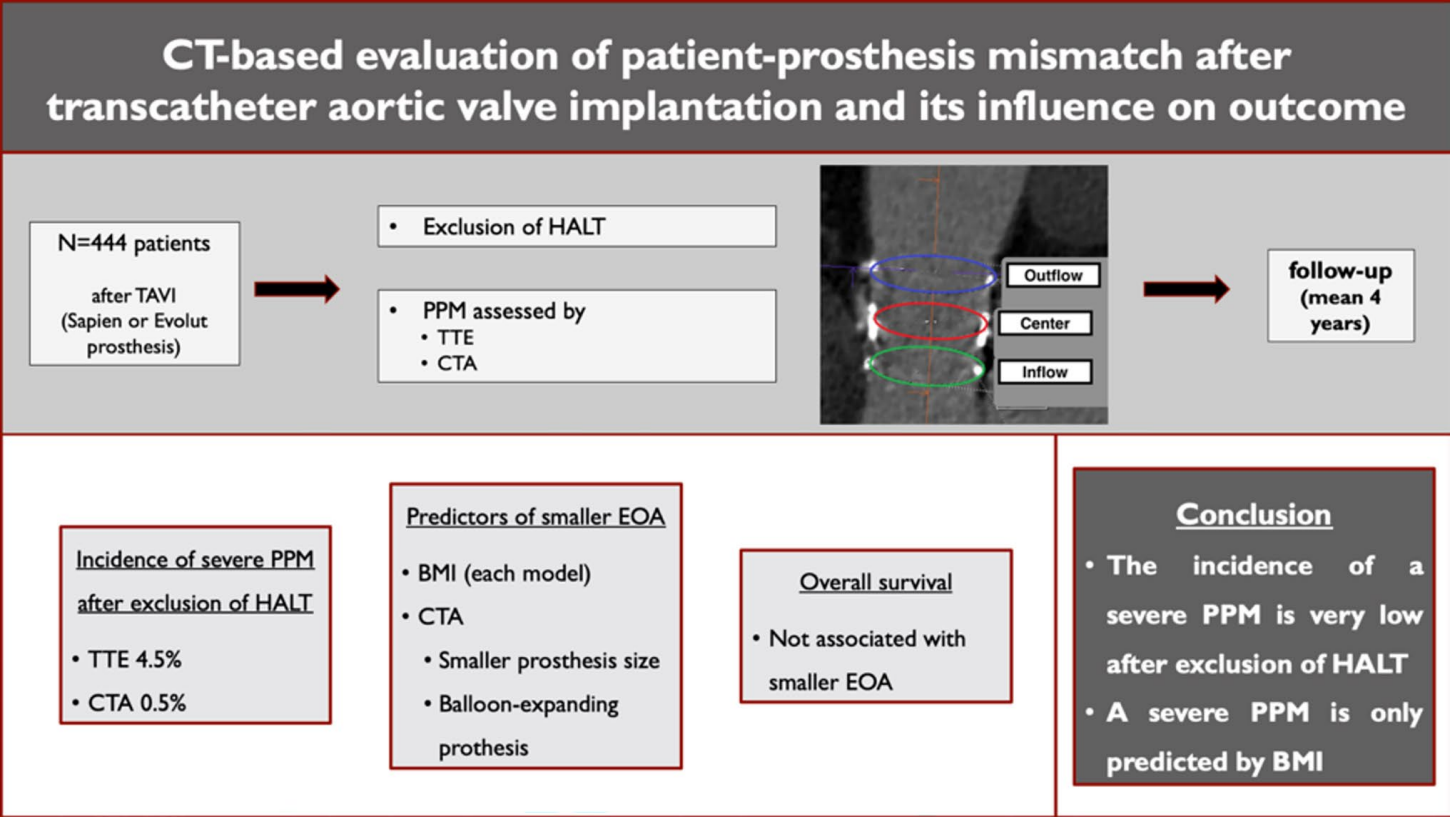

**Supplementary Information:**

The online version contains supplementary material available at 10.1007/s00392-025-02701-9.

## Introduction

Patient-prosthesis mismatch (PPM, calculated by continuity equation in echocardiography) after trans-catheter aortic valve implantation (TAVI) is a well-described risk factor for heart failure, re-hospitalizations and increased mortality in patients after TAVI [1]. It is described as an effective orifice area of the prosthetic heart valve that is too small in relation to the body size of the patient [2]. In general, PPM is less common after TAVI compared to surgical valve replacement [3]. Yet, the incidence is described to be between 10 and 45% overall [4, 5] and up to 10% [4] regarding a severe PPM. However, data on PPM influencing factors and outcome of patients with PPM are conflicting. As TAVI numbers are further increasing and indications are extending to younger patients, further research for prevention and management of PPM is needed.

Previous data indicate that transthoracic echocardiography (TTE) often tends to overestimate the severity of PPM by underestimation of left ventricular outflow tract (LVOT) diameter [4]. This study found significantly lower rates of PPM using a hybrid measurement of echocardiography and computed tomography angiography (CTA) (using CT-based LVOT diameter measurements in the continuity equation). In this study, there were no differences for outcome for PPM assessed either with echocardiographic alone or with CTA within a follow-up time of one year in 765 patients. Tough one might assume that there is a number of patients with early asymptomatic valve thrombosis (hypo-attenuated leaflet thickening, HALT) included in this collective and other studies in this field. These HALTs, only detectable with CTA, occur with an incidence of up to 15 percent after TAVI and can influence the pressure gradient [6–8]. Hence, the incidence of PPM could be overestimated by the presence of HALT. Furthermore, current studies lack information about stent expansion and configuration from a post-TAVI CTA.

The primary aim of this study is therefore to assess both methods (transthoracic echocardiography and CTA) for the incidence of PPM in a large patient cohort with an extent follow-up of more than 5 years. Patients with HALT in routine post-TAVI-CTA will be excluded. Furthermore, our study intents to evaluate possible anatomic predictors for PPM analyzing stent geometry in post-TAVI CTA in order to derive prevention strategies.

## Methods

### Study population

This observational study was approved by the institutional review board (ethical approval reference number 69/17, 04.07.2023) and complied with the Declaration of Helsinki. Candidates for study inclusions were all patients who received a routine multiphasic ECG-triggered contrast enhanced CTA before and after TAVI between 2014 and 2023 at our center. Reasons for performing CTA post-TAVI were as previously described [8]. In brief, until routine post-TAVI, CTA was scheduled for each patient in accordance with the guidelines for thoracic aortic stent implantation [9], in order to identify possible complications such as aortic injury. In cases of patient refusal, severe renal failure, logistical reasons, or markedly reduced general health status, CTA was not performed. Each patient provided written informed consent prior to undergoing CTA.

Patients with HALT are excluded from the analysis. Implanted valve types were the self-expanding Evolut (Evolut Pro, Evolut Pro Plus or Evolut R, Medtronic, Minneapolis, MN, USA) and the balloon-expandable Sapien (Sapien 3 Ultra, Sapien XT or Sapien S3, Edwards Lifesciences Corp., Irvine, CA, USA). All echocardiographic, computed tomographic angiographic and clinical data were obtained from our institutional database. In our institution, all TAVI patients are monitored using a standardized follow-up protocol including contacts with questionnaire or telephone calls 30 days, 1 year and then yearly after procedure. All patients gave their written informed consent for the anonymized use of clinical, procedural and follow-up data at the time of the intervention.

### Pre- and post-TAVI-CTA

**Fig. 1:**
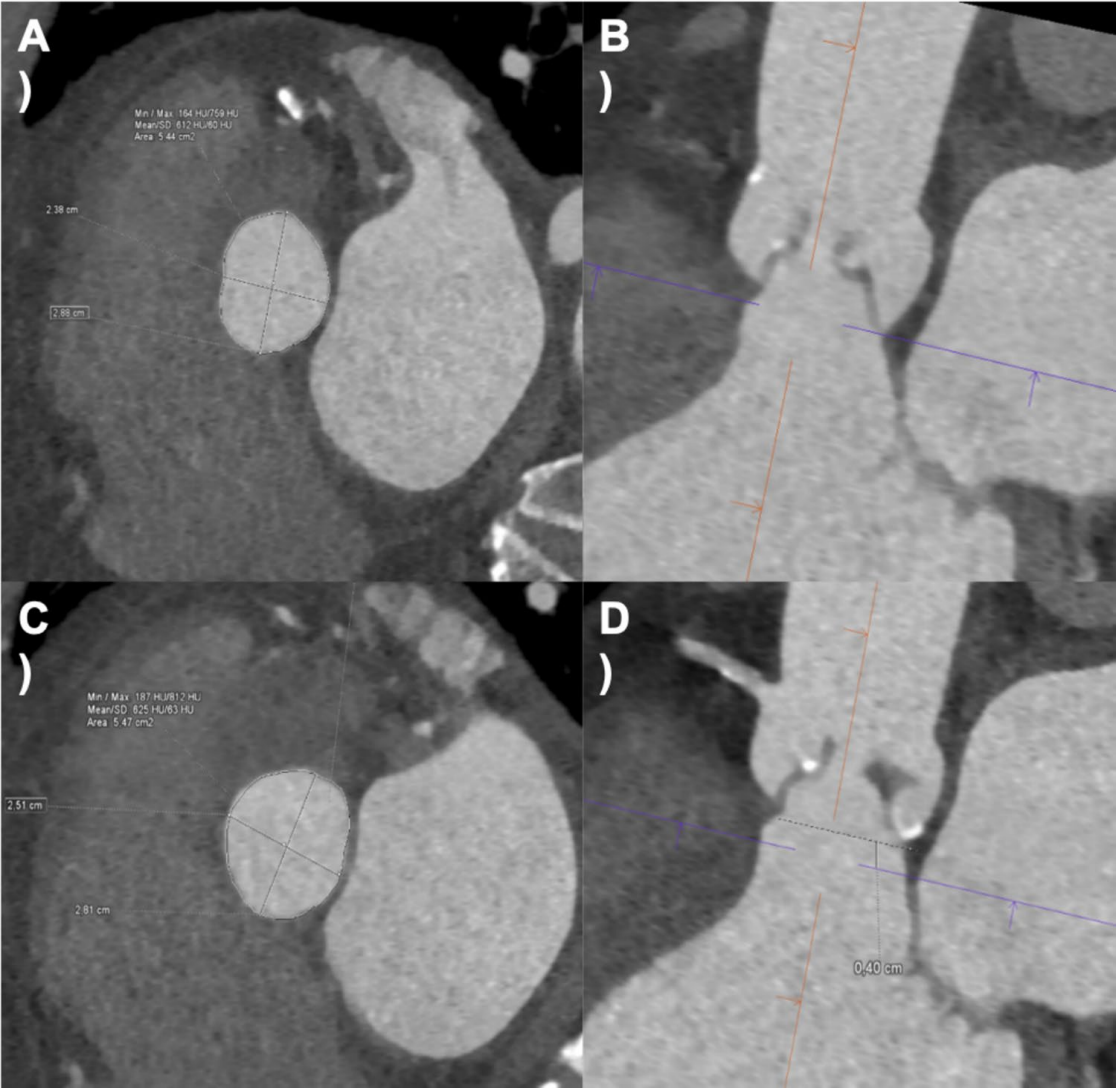
Pre-TAVI-CTA measurements. The figure shows the CTA measurement of aortic annulus area (**A**, **B** minimal and maximal diameter) and measurement of LVOT area (**C**, **D** minimal and maximal diameter) 4 mm below the aortic annulus. *CTA* computed tomography angiography, *LVOT* left ventricular outflow tract, *TAVI* trans-catheter aortic valve implantation

**Fig. 2:**
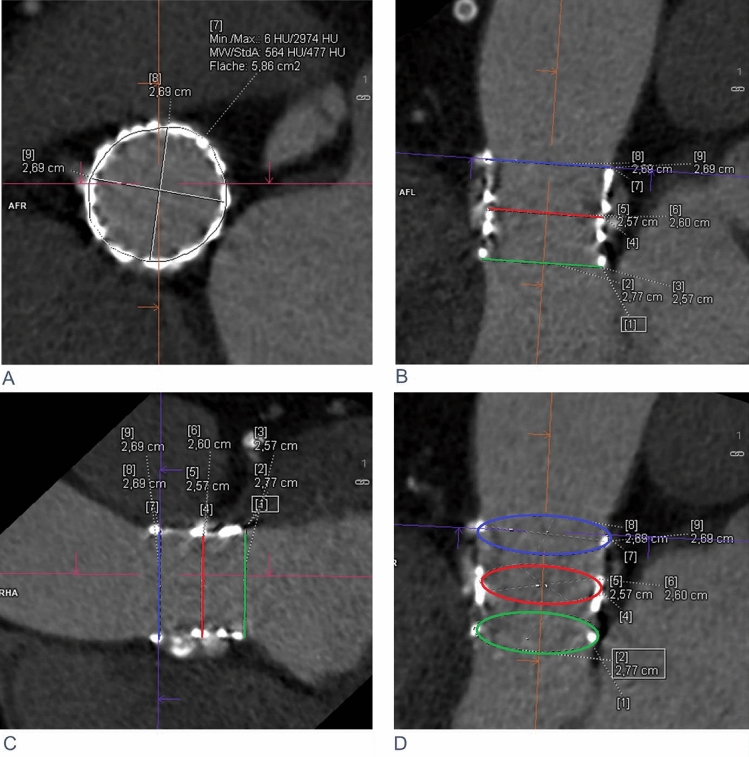
Post-TAVI-CTA measurements. The figure shows the post-TAVI-CTA measurements of a balloon-expanding prosthesis—the area and minimal and maximal diameter at the LVOT end (inflow, green), the stent center (center, red) and the aortic end (outflow, blue). *CTA* computed tomography angiography, *LVOT* left ventricular outflow tract, *TAVI* trans-catheter aortic valve implantation

Contrast-enhanced ECG-gated CTA was performed with a dual-source CT scanner (Siemens healthineers, Forchheim, Germany) as described previously [6]. Reconstruction for post-TAVI-CTA was conducted using a stent-specific reconstruction kernel (B46f). CTA images were analyzed in multi-planar reconstruction images using a post-processing workstation (Syngo Multimodality Workplace, Siemens Healthineers, Forchheim, Germany) by two experienced readers blinded to the clinical data (P.B. and P.R. with > 5 years of experience in CTA and both certified with the highest degree of cardiac CTA of the German Cardiac Society) independently. In the pre-TAVI-CTA, we measured the aortic annulus area as well as the LVOT area (defined 4 mm below the aortic annulus). In the post-TAVI-CTA, we measured the TAVI prosthesis area at the LVOT end (inflow), the stent center (center) and the aortic end (outflow), as well as the minimal and maximal diameter of the stent at each position respectively (Figs. 1, 2).

We defined an eccentricity index as maximal diameter stent center/minimal diameter stent center. Asymmetric expansion was calculated as [(area stent entry + area stent exit)/(2 × area stent center) − 1] × 100.

### PPM assessment

For the entire collective, the presence of PPM was assessed by the following methods:

- PPM calculated according to the continuity equation from TTE measurements using the LVOT from pre-TAVI TTE (defined as TTE-PPM).
- PPM calculated according to the continuity equation from TTE measurements using the LVOT from pre-TAVI CTA (defined as CT-PPM).

First, PPM in patients with an indexed effective orifice area (EOA) of > 0.85 cm^2^/m^2^ was defined as insignificant PPM, EOA of < 0.85–0.64 cm^2^/m^2^ as moderate PPM and EOA of ≤ 0.65 cm^2^/m^2^ as severe [1].

Second, PPM was defined according to body mass index (BMI) as recommended in literature [10]: in patients with BMI < 30 kg/m^2^ with an EOA of > 0.85 cm^2^/m^2^ as insignificant PPM, EOA of 0.85–0.64 cm^2^/m^2^ as moderate PPM and EOA of ≤ 0.65 cm^2^/m^2^ as severe PPM. In patients with BMI ≥ 30 kg/m^2^ with an indexed EOA of > 0.70 cm^2^/m^2^ as insignificant PPM, EOA of 0.70–0.56 cm^2^/m^2^ as moderate PPM and EOA of ≤ 0.55 cm^2^/m^2^ as severe PPM.

### Statistical analysis

Statistical analysis was performed using SPSS software Version 29.0.0.0 (IBM Corp., Armonk, NY, USA) and Stata (StataCorp LCC, Texas, USA, version 18). Categorical variables are expressed as frequencies and percentages, continuous variables as mean + standard deviation (SD) or median and interquartile range (IQR). Normal distribution was tested using Kolmogorov–Smirnov test. Comparison between two groups was achieved using *χ*^2^ test (categorical variables), Student’s *t* test (normal distributed continuous variables) or Mann–Whitney *U* test (non-normal distributed continuous variables). For possible predictors for PPM, linear multivariable regression models on the endpoint EOA were conducted for each PPM model. Survival was visualized using Kaplan–Meier survival estimate. Predictors for survival were analyzed using uni-variable and multivariable Cox regression models on the endpoint overall survival. A *p* value < 0.05 was considered statistically significant.

## Results

### Study population

670 patients after TAVI with evaluable pre-TAVI-CTA and post-TAVI CTA were included in the analysis. 24 patients (3.6%) underwent valve-in-valve replacement and 156 patients (23.3%) showed signs of HALT and were thus excluded. Image quality was too poor in 33 patients, mostly due to motion artifacts and inadequate contrast, making evaluation of HALT impossible (4.9%). In 13 patients (1.9%), data were incomplete, for example, due to loss to follow-up.

**Fig. 3:**
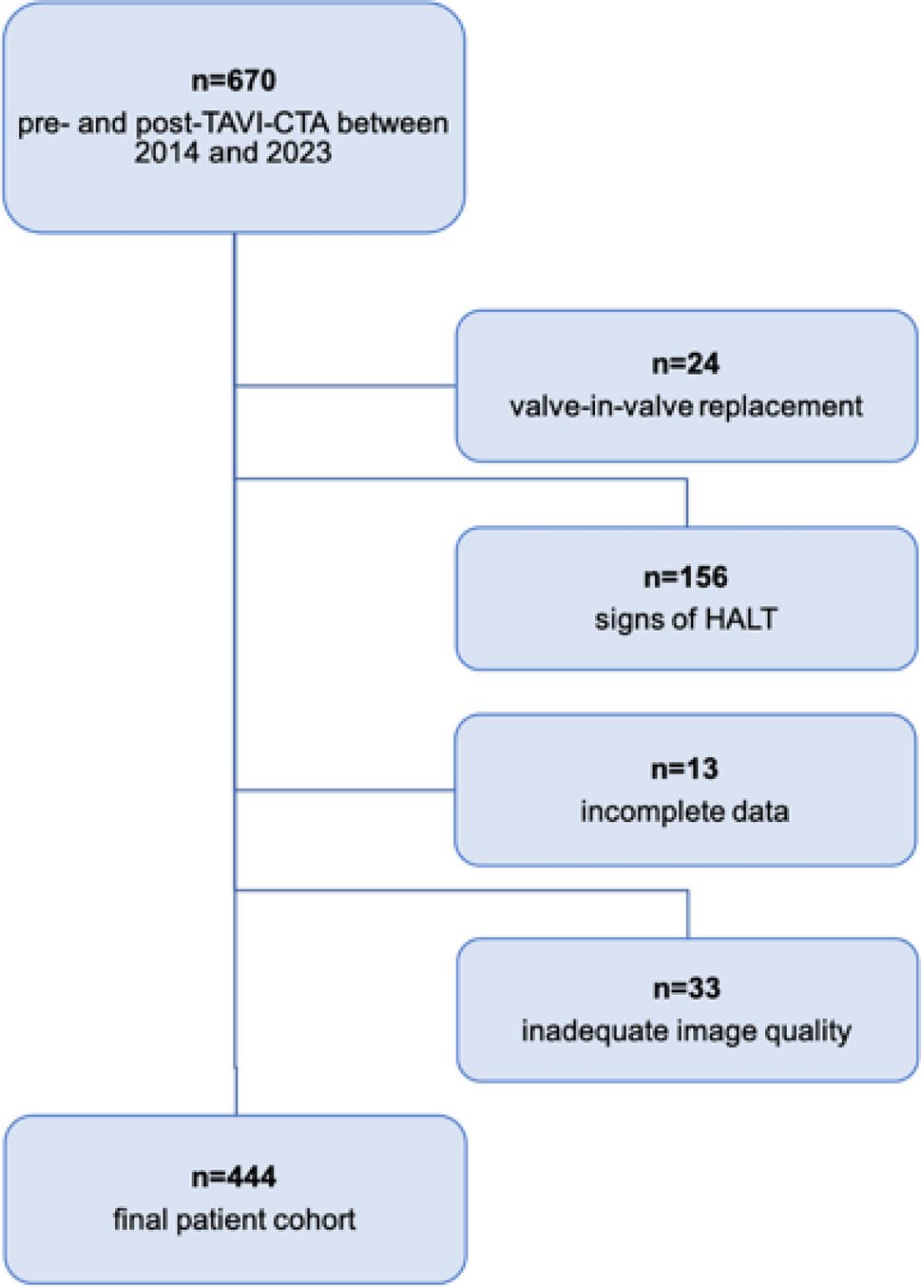
Flowchart of study enrollment. The figure shows a flowchart of the study enrollment. 670 patients were screened for inclusion in the study. After application of our exclusion criteria, 444 patients formed the final patient cohort. *CTA* computed tomography angiography, *HALT* hypo-attenuated leaflet thickening, *TAVI* trans-catheter aortic valve implantation

The remaining cohort consisted of 444 patients (49.3% female, 82.0 ± 5.3 years) (Fig. 3). All baseline characteristics are summarized in Table 1.

**Table 1:** Baseline characteristics of the study population

Patients	444
Age (years)	82.0 (± 5.3)
Sex	
Male	225 (50.7%)
Female	219 (49.3%)
BMI (kg/m^2^)	27.3 (± 4.7)
BAV	31 (6.8%)
Prosthesis type	
Evolut	103 (23.2%)
Sapien	341 (76.8%)
Prosthesis size (mm)	
20	1 (0.2%)
23	117 (25.6%)
26	204 (44.6%)
29	118 (25.8%)
31	3 (0.7%)
34	14 (3.1%)
Comorbidities	
Coronary artery disease	284 (64.0%)
Myocardial infarction	67 (15.1%)
Hypertension	368 (82.9%)
Diabetes mellitus	131 (29.5%)
Smoking history	71 (16%)

Values are mean ± standard deviation or frequencies and percentages

*BAV* bicuspid aortic valve, *BMI* body mass index

### Echocardiographic data and CTA measurements

The median pre-TAVI mean pressure gradient of the aortic valve of the entire patient cohort was 46.3 mmHg [45.0;47.5] and the median calculated aortic valve area 0.8 cm^2^ [0.7;0.8]. Post-TAVI mean pressure gradient was 10.0 mmHg [9.6;10.5]. All echocardiographic data are summarized in Table 2.

**Table 2:** Echocardiographic data of the study population

Pre-TAVI	
LVOT diameter TTE (mm)	21.3 [21.1; 21.5]
Maximum velocity LVOT (m/s)	0.9 [0.9; 1.0]
Mean pressure gradient aortic valve (mmHg)	46.3 [45.0; 47.5]
Maximum velocity aortic valve (m/s)	4.3 [4.3; 4.4]
Calculated aortic valve area TTE (cm^2^)	0.8 [0.7; 0.8]
Post-TAVI	
Maximum velocity LVOT (m/s)	1.2 [1.1; 1.2]
Maximum velocity TAVI (m/s)	2.1 [2.0; 2.1]
Mean velocity TAVI (m/s)	1.5 [1.4; 1.6]
Mean pressure gradient TAVI (mmHg)	10.0 [9.6; 10.5]
Maximum pressure gradient TAVI (mmHg)	18.7 [17.9; 19.5]

Values are median ± interquartile range

*LVOT* left ventricular outflow tract, *TAVI* trans-catheter aortic valve implantation, *TEE* trans-esophageal echocardiography, *TTE* transthoracic echocardiography

The median LVOT area in pre-TAVI CTA was 473.0 mm^2^ [462.8; 482.2] and the mean annulus area was 471.0 mm^2^ [462.8; 479.3]. Post-TAVI the median eccentricity index was 1.1 [1.0,1.1]. All CTA measurements are summarized in Table 3.

**Table 3:** CTA measurements

Pre-TAVI	
LVOT area (mm^2^)	473.0 [462.8; 482.2]
Maximum LVOT diameter (mm)	28.1 [27.8; 28.4]
Minimum LVOT diameter (mm)	21.1 [20.9; 21.4]
Aortic annulus area (mm^2^)	471.0 [462.8; 479.3]
Maximum annulus diameter (mm)	27.3 [27.0; 27.6]
Minimum annulus diameter (mm)	22.0 [31.7; 22.2]
Post-TAVI	
Area stent inflow (mm^2^)	436.2 [427.4; 444.9]
Maximum diameter stent inflow (mm)	24.8 [24.6; 25.1]
Minimum diameter stent inflow (mm)	22.3 [22.1; 23.6]
Area stent center (mm^2^)	398.2 [391.4; 404.9]
Maximum diameter stent center (mm)	23.5 [23.3; 24.7]
Minimum diameter stent center (mm)	21.6 [21.4; 21.8]
Area stent outflow (mm^2^)	511.6 [500.0; 523.2]
Maximum diameter stent outflow (mm)	26.1 [25.8; 26.4]
Minimum diameter stent outflow (mm)	24.6 [24.4; 25.9]
Asymmetric expansion	20.4 [18.5; 22.4]
Eccentricity index	1.1 [1.0; 1.1]

Values are median ± interquartile range

*LVOT* left ventricular outflow tract, *TAVI* trans-catheter aortic valve implantation

### Patient-prosthesis mismatch

A severe TTE-PPM was detected in 20 patients (4.5%) and a severe CT-PPM in 2 patients (0.5%). Comparison of the baseline characteristics between patients with no PPM and patients with severe PPM (assessed by the described methods) is shown in Table 4.

**Table 4:** Comparison of baseline characteristics between patients with severe PPM

	No TTE-PPM	Severe TTE-PPM	*p*-value	No CT-PPM	Severe CT-PPM	*p*-value
*N*	424	20	–-	442	2	–
Age	82.9 (79.0; 85.0)	81.0 (79.3; 84.8)	0.494	82 (79.0; 85.0)	82.0 (81.0; 82.0)	0.950
Sex			0.324			0.152
Male	218 (51.4)	8 (40.0)		226 (51.1)	0 (0.0)	
Female	206 (48.6)	12 (60.0)		216 (48.9)	2 (100.0)	
BMI	26.6 (24.1; 29.1)	28.6 (26.4; 32.6)	**0.013**	26.6 (24.3; 29.4)	27.5 (23.9; 27.5)	0.924
Prosthesis type			0.144			**0.011**
Evolut	103 (24.3)	2 (10.0)		103 (23.3)	2 (100.0)	
Sapien	321 (75.7)	18 (90.0)		339 (76.7)	0 (0.0)	
Prosthesis size (mm)			** < 0.001**			0.055
20	1 (0.2)	0 (0.0)		1 (0.2)	0 (0.0)	
23	103 (24.3)	12 (60.0)		114 (25.8)	2 (100.0)	
26	195 (46.0)	6 (30.0)		197 (44.6)	0 (0.0)	
29	111 (26.2)	2 (10.0)		115 (26.0)	0 (0.0)	
31	3 (0.7)	0 (0.0)		3 (0.7)	0 (0.0)	
34	11 (2.6)	0 (0.0)		12 (2.7)	0 (0.0)	
Asymmetric expansion	13.0 (8.8; 21.4)	13.3 (10.6; 18.0)	0.766	13.0 (8.8; 20.9)	61.4 (58.0; 61.4)	**0.031**
Eccentricity index	1.1 (1.0; 1.1)	1.0 (1.0; 1.1)	0.348	1.1 (1.0; 1.1)	1.1 (1.0; 1.1)	0.818

Values are median ± interquartile range or frequencies and percentages

Bold values represent the significance (=p-values) thus there are no other significance values available

*BMI* body mass index, *CT* computed tomography, *PPM* patient prosthesis mismatch, *TTE* transthoracic echocardiography

A calculation of linear regression analysis on the endpoint PPM had to be omitted due to small number of events. Instead body mass index (BMI) was identified as a predictor for the endpoint effective orifice area as surrogate for PPM in each assessment model (*p* < 0.001). Using the TTE-PPM and CT-PPM a smaller valve diameter was associated with a smaller effective orifice area (*p* < 0.001). Presence of a balloon-expandable prosthesis was associated with a smaller effective orifice area using the CT-PPM only (*p* = 0.033). Stent geometry did not influence the effective orifice area (*p* > 0.05 each method) (Table 5).

**Table 5:** Results of multivariable linear regression models on the endpoint effective orifice area for TTE-PPM (Model 1) and CT-PPM (Model 2)

	1	2
	0.054	0.072
Sex	[– 0.008, 0.115]	[– 0.010, 0.155]
	(0.087)	(0.085)
	0.002	0.003
Age	[– 0.002, 0.007]	[– 0.003, 0.009]
	(0.314)	(0.371)
	0.004	– 0.131
Prosthesis type	[– 0.084, 0.093]	[– 0.251,-0.011]
	(0.924)	(0.033)
	0.045	0.092
Prosthesis size	[0.031, 0.059]	[0.072, 0.111]
	**(< 0.001)**	**(< 0.001)**
	0.106	0.235
Eccentricity index	[– 0.229, 0.440]	[– 0.190, 0.661]
	(0.535)	(0.277)
	– 0.001	– 0.001
Asymmetric expansion	[– 0.003, 0.000]	[– 0.004, 0.001]
	(0.146)	(0.202)
	– 0.016	– 0.023
BMI	[– 0.021,-0.011]	[– 0.030,-0.016]
	**(< 0.001)**	**(< 0.001)**
	0.057	– 0.780
Constant	[– 0.619, 0.732]	[– 1.637, 0.077]
	(0.869)	(0.074)
Observations	444	444

Coefficients; 95% confidence intervals in brackets; *p* values in parentheses

Bold values represent the significance (=p-values) thus there are no other significance values available

*BMI* body mass index, *CT* computed tomography, *LVOT* left ventricular outflow tract, *PPM* patient prosthesis mismatch, *TTE* transthoracic echocardiography

### Follow-up

**Fig. 4:**
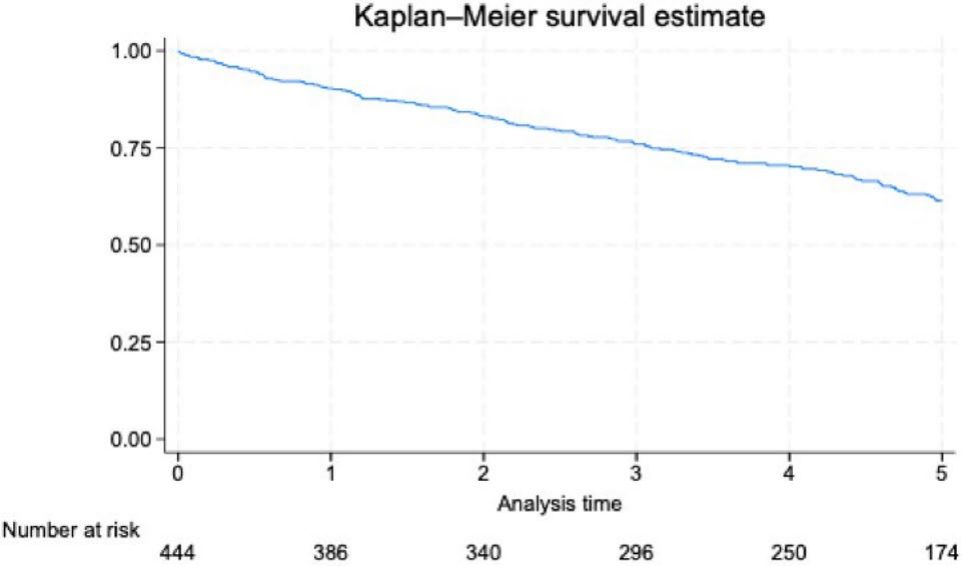
Kaplan–Meier survival estimate of the study cohort. The figure shows the Kaplan–Meier survival estimate of the study cohort. During the follow-up (mean 4 years), 161 patients (36.3%)

During the follow-up (mean 1485 days (4 years), IQR 749; 1847), 161 patients (36.3%) died (Fig. 4).

Uni-variable and multivariable Cox regression analyses showed no influence of a severe PPM on overall survival, irrespectively of the method (*p* > 0.05 each). (Table 6).

**Table 6:** Results of uni-variable (model 1–4) and multivariable (model 5–8) Cox regression models on the endpoint overall survival

	1	2	3	4	5	6	7	8
	0.732				0.654			
EOA TTE	[0.424; 1.263]				[0.361; 1.182]			
	(00.262)				(0.159)			
		1.563				2.031		
TTE-PPM		[0.732, 3.340]				[0.925, 4.462]		
		(0.249)				(0.078)		
			0.890				0.813	
EOA CT			[0.608; 1.303]				[0.523; 1.262]	
			(0.549)				(0.355)	
				5.509				6.206
CT-PPM				[0.764, 39.731]				[0.784, 49.107]
				(0.090)				(0.084)
					0.783	0.768	0.779	0.775
Sex					[0.533; 1.149]	[0.523, 1.128]	[0.531; 1.144]	[0.527, 1.139]
					(0.212]	(0.178)	(0.203)	(0.194)
					1.051	1.053	1.051	1.051
Age					[1.019; 1.084]	[1.021, 1.086]	[1.019; 1.084)	[1.019, 1.084]
					0.002	(0.001)	(0.002)	(0.002)
					0.749	0.759	0.729	0.732
Prosthesis					[0.412; 0.1363]	[0.417, 1.382]	[0.400; 1.328]	[0.401, 1.335]
Type					(0.344)	(0.368)	(0.302)	(0.309)
					0.988	0.978	0.988	0.978
Prosthesis					[0.903; 1.080]	[0.896, 1.067]	[0.899; 1.086]	[0.896, 1.067]
Size					(0.785)	(0.616)	(0.810)	(0.620)
					1.549	1.743	1.554	1.745
Eccentricity					[0.174; 13.776]	[0.195, 15.554]	[0.175; 13.794]	[0.196, 15.524]
Index					(0.695)	(0.619)	(0.692)	(0.618)
					1.006	1.006	1.006	1.006
Asymmetric					[0.995; 1.018]	[0.995, 1.017]	[0.995; 1.018]	[0.995, 1.017]
Expansion					(0.265)	(0.274)	(0.250)	(0.291)
					0.989	0.993	0.992	0.996
BMI					[0.952; 1.028)	[0.957, 1.031]	[0.954; 1.030]	[0.960, 1.033]
					(0.578)	(0.713)	(0.662)	(0.823)
Observations	444	444	444	444	444	444	444	444

*BMI* body mass index, *CT* computed tomography, *EOA* effective orifice area, *LVOT* left ventricular outflow tract, *PPM* patient prosthesis mismatch, *TTE* transthoracic echocardiography

Hazard Ratios; 95% confidence intervals in brackets; *p* values in parentheses

#### PPM in relation to BMI

Using lower cutoff values of EOA for PPM in obese patients, moderate and severe TTE-PPM were detected in 72 patients (16.2%) and CT-PPM in 11 patients (2.5%).

Regarding the analysis of PPM using lower cutoff values of EOA for PPM in obese patients, uni-variable and multivariable Cox regression analyses showed no influence on a moderate or a severe PPM on overall survival, irrespectively of the method (*p* > 0.05 each) (Table 7).

**Table 7:** Results of uni-variable (model 1–4) and multivariable (model 5–8) Cox regression models on the endpoint overall survival (BMI defined PPM)

	1	2	3	4	5	6	7	8
	0.732				0.654			
EOA TTE	[0.424, 1.263]				[0.361, 1.182]			
	(0.262)				(0.159)			
		1.411				1.497		
TTE-PPM		[0.931, 2.139]				[0.981, 2.284]		
		(0.104)				(0.061)		
			0.890				0.813	
EOA CT			[0.608, 1.303]				[0.523, 1.262]	
			(0.549)				(0.355)	
				2.294				2.658
CT-PPM				[0.844, 6.235]				[0.958, 7.377]
				(0.104)				(0.060)
					0.783	0.766	0.779	0.776
Sex					[0.533, 1.149]	[0.521, 1.126]	[0.531, 1.144]	[0.528, 1.140]
					(0.212)	(0.175)	(0.203)	(0.196)
					1.051	1.053	1.051	1.052
Alter					[1.019, 1.084]	[1.020, 1.086]	[1.019, 1.084]	[1.020, 1.085]
					(0.002)	(0.001)	(0.002)	(0.001)
					0.749	0.763	0.729	0.742
Prosthesis					[0.412, 1.363]	[0.421, 1.384]	[0.400, 1.328]	[0.409, 1.347]
Type					(0.344)	(0.373)	(0.302)	(0.326)
					0.988	0.977	0.988	0.979
Prosthesis					[0.903, 1.080]	[0.895, 1.066]	[0.899, 1.086]	[0.898, 1.068]
Size					(0.785)	(0.602)	(0.810)	(0.635)
					1.549	1.377	1.554	1.514
Eccentricity					[0.174, 13.776]	[0.152, 12.519]	[0.175, 13.794]	[0.169, 13.550]
Index					(0.695)	(0.776)	(0.692)	(0.711)
					1.006	1.007	1.006	1.006
Asymmetric					[0.995, 1.018]	[0.996, 1.018]	[0.995, 1.018]	[0.995, 1.017]
Expansion					(0.265)	(0.225)	(0.250)	(0.276)
					0.989	0.996	0.992	0.997
BMI					[0.952, 1.028]	[0.960, 1.033]	[0.954, 1.030]	[0.961, 1.034]
					(0.578)	(0.820)	(0.662)	(0.852)
Observations	444	444	444	444	444	444	444	444

*BMI* body mass index, *CT* computed tomography, *EOA* effective orifice area, *LVOT* left ventricular outflow tract, *PPM* patient prosthesis mismatch, *TTE* transthoracic echocardiography

Hazard Ratios; 95% confidence intervals in brackets; *p* values in parentheses

## Discussion

To our knowledge, this study represents one of the largest cohorts of patients undergoing CTA after TAVI with long-term follow-ups. This analysis is unique due to its exclusion of early asymptomatic valve thrombosis (HALT) in the evaluation of patient-prosthesis mismatch.

The main findings are as follows:

1. After exclusion of patients with HALT, the incidence of a severe PPM was lower than reported in previous studies (TTE assessment 4.5%).
2. Using a CTA-based assessment, the incidence of a severe PPM was even lower (0.5%).
3. Only BMI was a predictor for a smaller EOA in each assessment model.
4. A smaller EOA assessed by CTA was associated with a smaller valve diameter and presence of balloon-expanding prosthesis.
5. The incidence of a smaller EOA was not associated with stent geometry.
6. Overall survival of these patients was not significantly influenced by a smaller EOA.

### Assessment of PPM

The incidence of PPM after TAVI is described to be between 10 and 45% overall [4, 5] and up to 10% [4] regarding a severe PPM. The broad variability has been consistently documented across multiple studies in recent years and appears to be even more pronounced in the context of PPM following surgical valve replacement. An overview of the reported incidence of severe PPM in selected studies is provided in Supplementary Table 1.

The broad range of PPM incidence might be attributed to several factors. Previous studies indicate that PPM assessed by TTE resulted in a higher incidence compared to other assessment methods such as TEE or CTA assessments or predicted PPM [4, 5, 11–14]. These results were reproducible in our patient cohort. This is most likely attributed to underestimation of LVOT diameter in TTE which leads to smaller EOA using the continuity equation [15].

In our cohort, the incidence of severe PPM using a CTA-based assessment was even lower than previously described (0.5%). This might be driven by the fact that we exclude early asymptomatic valve thrombosis (HALT) of our cohort. These HALTs are only detectable with CTA with an incidence of up to 30 percent and can influence the pressure gradient and thereby the diagnosis of PPM [8, 16]. We therefore propose that a systematic exclusion of HALT is essential for evaluation of PPM to exclude this bias.

### Predictors of PPM

Previous studies already investigated predictors of PPM to develop potential prevention strategies. In our patient cohort, only BMI was a predictor for a smaller EOA in each assessment model. This is in line with previous studies [17, 18] and might be attributed to an overestimation of PPM in obese patients due to the use of the body surface area for normalization of EOA [19].

A smaller EOA assessed by TTE and CTA was furthermore associated with a smaller valve diameter and a smaller EOA assessed by CTA with the presence of balloon-expanding prosthesis. This is also in line with previous studies [17, 18, 20–24].

To date, there were no studies analyzing stent geometry after TAVI for PPM development and subsequently evaluate possible anatomic predictors. In our patient cohort receiving post-TAVI CTA, neither eccentricity nor asymmetric expansion of the valve was associated with a smaller EOA. Thus, performance of TAVI procedures as established (normal valve position and configuration) does not influence the development of PPM and does not require adjustments.

### Outcome of patients with PPM

Data on outcome of patients with PPM are conflicting. A recent meta-analysis showed that severe PPM, not moderate PPM, was associated with a higher risk of mortality regarding a follow-up period of up to 5 years [25]. However, there are also multiple studies reporting no evidence of an association of PPM with overall survival [17, 18, 20, 26, 27]. These differences might be attributed to the different assessment methods of PPM, as described above, and large differences regarding the follow-up period of the studies.

In our patient cohort with an extent follow-up of about 5 years, overall survival was not significantly influenced by a smaller EOA assessed by TTE or CTA.

Previous studies using CTA assessment of PPM showed similar conflicting results as the overall literature regarding PPM: Fukui et al. reported an association of PPM with mortality [13], while Mooney et al. and Sugiyama et al. showed no association of PPM with mortality [4, 5].

Overall, further research regarding the outcome of patients with PPM is needed, especially with consideration of the different assessment methods and, in our opinion, after systematic exclusion of HALT.

### Limitations

Several limitations must be addressed. First, we report on a retrospective study and the findings need external validation in further studies. The small number of patients with PPM after exclusion of HALT limits the statistical power of the analyses and prevents further analyses with adjustments for baseline variables e.g., exact valve sizes and sub-analyses. We thus cannot exclude minor associations. A calculation of linear regression analysis on the endpoint PPM was not possible due to small number of events. Thus, predictors for the endpoint effective orifice area as surrogate for PPM were assessed.

Despite these limitations, the impact of our study is strengthened by the unique exclusion of patients with HALT regarding the assessment of PPM.

## Conclusions

Our study detected a very low incidence of severe PPM after exclusion of patients with HALT using a TTE- and CTA-based assessment. Only BMI was a predictor for a smaller EOA in each assessment model. The incidence of a smaller EOA was not associated with stent geometry. Overall survival of these patients was not significantly influenced by a smaller EOA.

Our findings highlight the importance of exclusion of HALT in TAVI patients regarding the assessment of PPM and the need for further research regarding influencing factors and outcome of patients with PPM.

***Clinical perspective*** In our patient cohort, the incidence of a severe patient-prosthesis mismatch (PPM) after trans-catheter aortic valve implantation (TAVI) was very low once hypo-attenuated leaflet thickening (HALT) was excluded. We therefore conclude that the exclusion of HALT is a crucial step in assessing PPM and should be performed before evaluating further therapeutic options for patients with PPM. We found body mass index (BMI) to be the only significant influencing factor in the development of PPM across all assessment models. However, further research is needed to better understand additional influencing factors and the overall impact of PPM on patient outcomes.

## Supplementary Information

Below is the link to the electronic supplementary material.

Supplementary file1 (DOCX 26 KB)

## Data Availability

The data are not publicly available due to containing information that could compromise the privacy of the research participants.

## Abbreviations

BMI: Body mass index
CTA: Computed tomography angiography
EOA: Effective orifice area
HALT: Hypo-attenuated leaflet thickening
IQR: Interquartile range
LVOT: Left ventricular outflow tract
PPM: Patient-prosthesis mismatch
SD: Standard deviation
TAVI: Trans-catheter aortic valve implantation
TTE: Transthoracic echocardiography
