# Supplementary material for: CT-based evaluation of patient-prosthesis mismatch after transcatheter aortic valve implantation and its influence on outcome
Source: Clin Res Cardiol. 2025 Jun 23;115(7):1154–65. doi: 10.1007/s00392-025-02701-9 (PMC13249650; doi:10.1007/s00392-025-02701-9)
Supplement: Supplementary file 1 — Supplementary file1 (DOCX 26 KB) [file 392_2025_2701_MOESM1_ESM.docx]

| **Study** | **Procedure** | **Number of patients** | **Incidence of severe PPM** |
| --- | --- | --- | --- |
| Pibarot et al, 2014 [1] | Surgical valve replacement  TAVI | 270  304 and 1.637 | 28.1%  19.7% and 13.6% |
| Thyregod et al, 2016 [2] | Surgical valve replacement  TAVI | 135  145 | 33.9%  14.0% |
| Mooney et al, 2017 [3] | TAVI | 765 | 6% and 9% |
| Fallon et al, 2018 [4] | Surgical valve replacement | 59.779 | 11% |
| Herrmann et al, 2018 [5] | TAVI | 62.125 | 12% |
| Schofer et al, 2019 [6] | TAVI | 1.309 | 12.9% |
| Ternacle et al, 2021 [7] | Surgical vale replacement  TAVI | 929  1.069 | 23.6%  5.7% |
| Yamanaka et al, 2025 [8] | TAVI | 7.051 | 1.3% |
| Nam et al, 2025 [9] | Surgical valve replacement | 10.607 | 2% |

TAVI – transcatheter aortic valve implantation

**References**

1. Pibarot P, Weissman NJ, Stewart WJ, et al (2014) Incidence and sequelae of prosthesis-patient mismatch in transcatheter versus surgical valve replacement in high-risk patients with severe aortic stenosis: a PARTNER trial cohort--a analysis. J Am Coll Cardiol 64:1323–1334. https://doi.org/10.1016/j.jacc.2014.06.1195

2. Thyregod HGH, Steinbrüchel DA, Ihlemann N, et al (2016) No clinical effect of prosthesis-patient mismatch after transcatheter versus surgical aortic valve replacement in intermediate- and low-risk patients with severe aortic valve stenosis at mid-term follow-up: an analysis from the NOTION trial. Eur J Cardio-Thorac Surg Off J Eur Assoc Cardio-Thorac Surg 50:721–728. https://doi.org/10.1093/ejcts/ezw095

3. Mooney J, Sellers SL, Blanke P, et al (2017) CT-Defined Prosthesis-Patient Mismatch Downgrades Frequency and Severity, and Demonstrates No Association With Adverse Outcomes After Transcatheter Aortic Valve Replacement. JACC Cardiovasc Interv 10:1578–1587. https://doi.org/10.1016/j.jcin.2017.05.031

4. Fallon JM, DeSimone JP, Brennan JM, et al (2018) The Incidence and Consequence of Prosthesis-Patient Mismatch After Surgical Aortic Valve Replacement. Ann Thorac Surg 106:14–22. https://doi.org/10.1016/j.athoracsur.2018.01.090

5. Herrmann HC, Daneshvar SA, Fonarow GC, et al (2018) Prosthesis-Patient Mismatch in Patients Undergoing Transcatheter Aortic Valve Replacement: From the STS/ACC TVT Registry. J Am Coll Cardiol 72:2701–2711. https://doi.org/10.1016/j.jacc.2018.09.001

6. Schofer N, Deuschl F, Rübsamen N, et al (2019) Prosthesis-patient mismatch after transcatheter aortic valve implantation: prevalence and prognostic impact with respect to baseline left ventricular function. EuroIntervention J Eur Collab Work Group Interv Cardiol Eur Soc Cardiol 14:1648–1655. https://doi.org/10.4244/EIJ-D-18-00827

7. Ternacle J, Pibarot P, Herrmann HC, et al (2021) Prosthesis-Patient Mismatch After Aortic Valve Replacement in the PARTNER 2 Trial and Registry. JACC Cardiovasc Interv 14:1466–1477. https://doi.org/10.1016/j.jcin.2021.03.069

8. Yamanaka F, Shishido K, Moriyama N, et al (2025) Incidence and Prognosis of Prosthesis-Patient Mismatch After Transcatheter Aortic Valve Replacement for Bicuspid Aortic Stenosis. JACC Cardiovasc Interv 18:492–502. https://doi.org/10.1016/j.jcin.2024.10.002

9. Nam L, Singh R, Hirji SA, et al (2025) A Multi-Institutional Study on the Prevalence and Clinical Impact of Patient-Prosthesis Mismatch in Surgical Aortic Valve Replacement. Ann Thorac Surg S0003-4975(25)00421–7. https://doi.org/10.1016/j.athoracsur.2025.04.036
